# Rhythmic auditory stimulation promotes gait recovery in Parkinson's patients: A systematic review and meta-analysis

**DOI:** 10.3389/fneur.2022.940419

**Published:** 2022-07-28

**Authors:** Xiaofan Ye, Ling Li, Rong He, Yizhen Jia, Waisang Poon

**Affiliations:** ^1^Neuromedicine Center, University of Hong Kong Shenzhen Hospital, Shenzhen, China; ^2^Physiotherapy Department, University of Hong Kong Shenzhen Hospital, Shenzhen, China; ^3^Core Laboratory, University of Hong Kong Shenzhen Hospital, Shenzhen, China

**Keywords:** Parkinson's patients, rhythmic auditory stimulation, gait, mobility, meta-analysis

## Abstract

**Objective:**

Using rhythmic auditory stimulation (RAS) to improve gait disturbance in Parkinson's disease (PD) is an available treatment option, yet a consensus on its effectiveness remains controversial. We summarized the effects of RAS on gait, functional activity and quality of life in PD patients through a systematic review and meta-analysis.

**Methods:**

PubMed, Embase, Web of Science, Medline, and Cochrane Library databases were initially searched to identify relevant literature up to August 2021. Next, the methodological quality of eligible comparative studies was assessed by the Physiotherapy Evidence Database Scale. The treatment effects to clinical outcome in relation to gait, motor activities, and quality of life were analyzed.

**Results:**

A total of 18 studies consisted of 774 subjects were included in this meta-analysis. Comparing with the control group, RAS had significantly increased stride length (*p* < 0.001), accelerated gait speed (*p* < 0.001), reduced the occurrence of freezing events during walking (*P* = 0.009), achieved an improvement in Unified Parkinson's Disease Rating Scale (UPDRS) II (*P* = 0.030), UPDRS-III (*P* < 0.001) and Parkinson's Disease Quality of Life Questionnaire (PDQL) (*p* = 0.009) scores over an interval of 1–26 months.

**Conclusion:**

In this meta-analysis of 18 randomized controlled trials, we have demonstrated that RAS improves the general motor functions (UPDRS-III), particularly in gait, mobility and quality of life, in patients with Parkinson's disease.

## Introduction

Parkinson's disease (PD) is a common age-related neurodegenerative disease after Alzheimer's disease, affecting 1% of the world's population over the age of 60 years (1). With an aging population, the number PD patients are expected to reach 13 million by 2024, doubling in the next 10 years by 2034 (2–4). PD patients often present with tremor, rigidity, bradykinesia, gait disturbance, balance and coordination disorders, accompanied by non-motor-related symptoms such as cognitive and psychological impairment, neurobehavioral abnormalities, and sleep disturbances (5–7). Motor symptoms are caused by the loss and degeneration of dopaminergic neurons in the dense part of substantia nigra. As there is no curative treatment for Parkinson's disease, symptomatic relief by medications and the Deep Brain Stimulation are regarded as the main management modalities (8). Pharmacological interventions are primarily to increase dopamine levels *via* the use of dopaminergic drugs. However, long-term use of dopaminergic drugs can have serious side effects on patients, such as loss of efficacy and accumulation of toxicity (9, 10). Besides, the axial symptoms of gait disturbances do not respond to pharmacotherapy and deep brain stimulation (11–14). 25–60% of patients experience freezing of gait usually after several years from disease-onset. As Gait disturbances respond poorly to treatments, physical rehabilitation techniques are gaining interest as an adjunct in the management of these patients when the combined therapies of medication and surgery are failing (5, 15, 16).

Physical activity has a positive impact on gait, cognitive function, and quality of life in patients with Parkinson's disease (17, 18). The joy of an independent functional mobility does generate a positive motivation in these patients (19). Music is an effective emotional relaxant that helps relieve anxiety and pain (20). Rhythmic auditory and visual cues can improve all types of freezing of gait, dopamine-responsive or dopamine-resistant, according to the literature, a Level B evidence (4). At present, there is no systematic review or meta-analysis using high quality randomized double-blinded controlled trials of sufficient size and power to lead to a definitive study in the future (4). Therefore, combining music with physical activity is a feasible, enjoyable, and probably sustainable option. Studies have shown that gait training accompanied by music and rhythmic auditory stimulation (RAS) can significantly increase patients' stride length and speed (21). Compared with treadmill gait training alone, treadmill gait training with rhythmic auditory stimulation can significantly improve gait and quality of life (22, 23). Several systematic reviews and meta-analyses have reported the effectiveness of RAS on gait in patients with Parkinson's disease (24, 25). In addition to the retrospective cohort studies, there have been several recent randomized controlled trials (RCTs) in the field. We have updated the published RCTs with a more comprehensive meta-analysis.

## Methods

### Study design

This systematic review and meta-analysis were carried out under the statement of Preferred Reporting Items for Systematic Reviews, PRISMA (26).

### Retrieval strategy and literature selection

PubMed, Embase, Web of Science, Medline, and Cochrane Library databases were thoroughly searched, to obtain studies published between January 2000 and August 2021. The searching keywords included (“tread,” “gait,” “train,” “exercise,” “rehabilitation” or “treatment”) and (“Rhythmic,” “auditory stimulation,” “musical stimulation,” “music” or “acoustic”) and (“Parkinson's disease”).

All eligible studies in this meta-analysis had to meet the following criteria: (1) patients had idiopathic Parkinson's disease; (2) patients in the intervention group received a course of music or rhythmic auditory stimulation during physical therapy whereas the control group received conventional physical therapy; (3) the effects of the intervention on gait, mobility, and quality of life were reported; (4) patients participating in study ≥ 10; (5) the study should be publish in English and peer-reviewed journals; (6) the study should be randomized controlled trials.

Exclusion criteria: (1) patients should not be too frail to receive physical therapy. They should not be cognitively impaired to follow instructions of physical therapy; (2) case reports, reviews, letters, comments and abstracts were not included; (3) studies where assessment outcome was unavailable.

### Quality evaluation

The Physiotherapy Evidence Database (PEDro) scale was used to assess the quality of included studies (27). The PEDro scale consists of 11 items, including random assignment, undercover assignment, baseline comparability, subject blinding, therapist blinding, assessor blinding, adequate follow-up, intention-to-treat analysis, between-group comparisons, point measures, and variance measures. The maximum PEDro score is 10. The quality of the study was classified as “excellent” (9–10 points), “good” (6–8 points), and “fair” (≤ 5 points) based on the PEDro score (25). Studies with a PEDro score ≥ 6 will be included in this meta-analysis. The quality assessment was performed independently by two researchers (LL and HR). When any disagreements arose, the two researchers resolved them in discussion with a third researcher (YXF).

### Data extraction

Two researchers (LL and HR) independently extracted primary data from eligible studies using a standardized form. The following relevant variables would be extracted: (1) study characteristics: first author, year of publication, region of study, and PEDro score; (2) subject characteristics: sample size, age, disease duration, and Hoehn and Yahr staging; (3) gait kinematic parameters: stride length, stride duration, gait speed, stride frequency, swing, and timed up-and-go test (TUG); and (4) clinical parameters: Unified Parkinson's Disease Rating Scale (UPDRS), Parkinson's Disease Quality of Life Questionnaire (PDQL) score, Berg Balance Scale (BBS), Falls Efficacy Scale (FES), and Freezing of Gait Questionnaire (FOGQ). UPDRS-III is the most popular assessment tool for motor function impairment for patients with Parkinson's disease. We have therefore chosen it as the primary outcome for our study.

Both freezing of gait (FoG) and Speed are regarded as refractory symptoms in advanced Parkinson's disease. We have selected them for secondary outcomes (FOGQ and Speed). If the corresponding data could not be extracted directly from the study, it would need to be reanalyzed. Where there were disagreements between the above two researchers, a third researcher (YXF) was asked to review literatures until a consensus was reached. The data management and statistical analysis were performed by YXF and reviewed by statistician JYZ from the core laboratory.

### Data analysis

Analyses were conducted using STATA 16.0 SE. As the outcomes investigated were continuous variables and scale of measurement, we used mean difference (MD) and corresponding 95% confidence interval (95% CI) for assessment. When the same scale and units were used for all study outcomes, the weighted mean difference (WMD) and its corresponding 95% CI were used as the pooled statistic in the meta-analysis. Forest plots were used to display the pooled results of the meta-analysis. Between-study heterogeneity was assessed using *I*^2^ and two-tailed *p*-values (28). No statistically significant heterogeneity was considered when *I*^2^ < 50%, *p* > 0.05, so a fixed-effects model was adopted. Otherwise, a random-effects model was applied (29). The effect size was significant when the pooled 95% CI excluded 0 and the *p*-value < 0.05.

## Results

**Figure 1:**
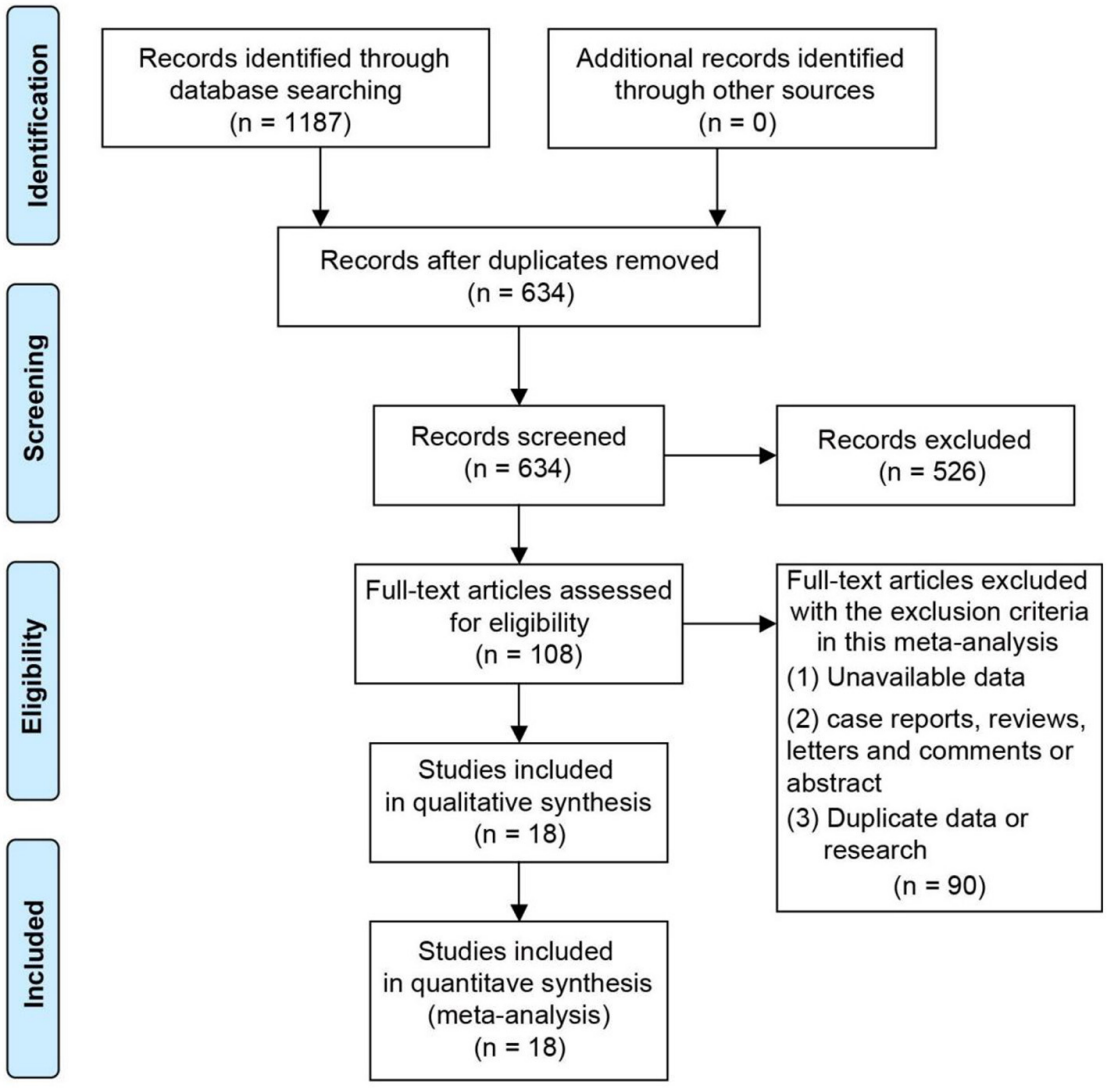
Flow chart of the search and screening of the included literature.

Flow chart Figure 1 showed the process and results of literature screening. By searching the online database, a total of 1,187 studies were obtained. After excluding the duplicate studies, 634 remained. Five hundred and twenty six studies were removed as they did not meet the eligibility criteria. Finally, 18 studies were included after the full text of 108 literature were read (22, 23, 30–45).

A total of 774 subjects were included in these 18 studies, there were a total of 396 patients in the intervention group and 378 patients in the control group. The sample sizes of subjects included in these studies ranged from 16 to 112. The mean age of the participants in each study ranged from 62 to 72 years. The regions of eligible studies included Italy (22, 33, 36, 38, 42, 44, 45), Sweden (23, 31), Poland (39), Brazil (34) and Romania (30), Canada, (32, 43), the United States (35, 40, 41), China (37). The average disease duration of PD patients included in the study ranged from 4 to 13 years. Three papers did not specify the duration of disease in the included subjects. The Hoehn-Yahr staging of the included Parkinson's patients covered stages 1 to 4. Similarly, three studies did not specify the Hoehn-Yahr staging of the included subjects. The PEDro scores of the included studies were all six and above. All eligible studies were RCT studies. Table 1 demonstrates the essential characteristics of all eligible studies.

**Table 1:** Main characteristics of the studies included in the meta-analysis.

**References**	**Region**	**Total sample size**	**Age**	**Disease duration**	**Hoehn-Yahr stage**	**PEDro score**
Pacchetti et al. (45)	Italy	32	62.85 ± 4.93	5.00 ± 2.52	2–3	8
Frazzitta et al. (44)	Italy	40	71.00 ± 7.42	13.05 ± 4.30	3	7
de Bruin et al. (43)	Canada	22	65.55 ± 6.47	5.45 ± 3.81	2–3	7
Modugno et al. (42)	Italy	20	62.60 ± 4.27	9.70 ± 4.60	2–4	7
Kadivar et al. (41)	USA	16	71.90 ± 6.20	NA	2–4	8
Pohl et al. (23)	Sweden	18	68.20 ± 5.10	8.80 ± 3.80	NA	6
Harro et al. (40)	USA	20	66.10 ± 10.31	4.12 ± 2.26	1–3	6
Song et al. (37)	China	112	65.90 ± 7.97	6.80 ± 2.99	NA	8
De Icco et al. (38)	Italy	35	74.00 ± 7.41	10.34 ± 4.60	2–4	7
Bukowska et al. (39)	Poland	55	63.42 ± 10.10	6.07 ± 4.11	2–3	7
Murgia et al. (36)	Italy	38	68.20 ± 10.51	6.35 ± 5.76	1–3	7
Thaut et al. (35)	USA	60	71.94 ± 7.47	11.04 ± 5.43	3–4	8
Calabro et al. (22)	Italy	50	71.50 ± 8.06	9.65 ± 2.99	2–3	7
De Luca et al. (33)	Italy	40	63.20 ± 8.40	NA	2–3	6
Pohl et al. (31)	Sweden	46	70.00 ± 6.52	6.35 ± 4.05	1–3	8
Mosabbir et al. (32)	Canada	36	69.40 ± 9.50	6.50 ± 4.40	NA	8
Capato et al. (34)	Brazil	102	72.75 ± 8.84	7.44 ± 6.91	1–3	7
Fodor et al. (30)	Romania	32	66.35 ± 5.66	NA	1–3	6

### Effect of RAS on gait parameters

**Figure 2:**
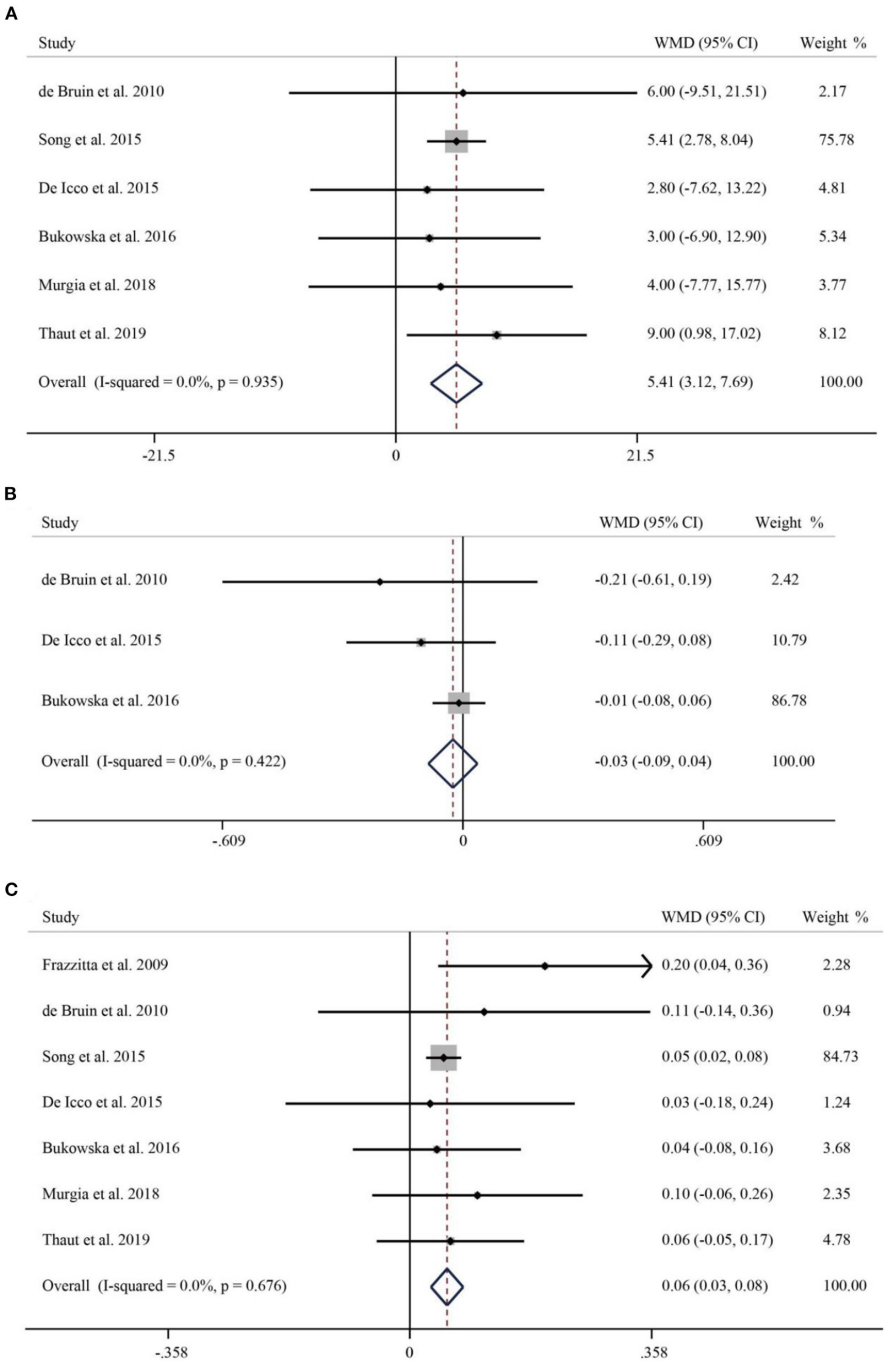
Forest plot of RAS vs. the control group for stride length **(A)**, stride duration **(B)**, and speed **(C)**.

A total of six studies reported the effect of RAS on the stride length of Parkinson's patients. As no significant heterogeneity was found (*I*^2^ = 0.0%, *p* = 0.935), we used a fixed-effects model for analysis. The stride length of patients in the intervention group significantly increased by 5 cm compared with the control group (WMD = 4.64, 95% CI: 3.12–7.69, *p* < 0.001) (Figure 2A). Three studies with a total of 112 patients reported on the stride duration of patients. The pooled WMD was −0.03 (95% CI: −0.09–0.04, *p* = 0.426), suggesting no significant effect of RAS on shortening stride duration (Figure 2B). Seven studies of published material on stride speed showed that rhythmic auditory stimulation significantly accelerated speed in patients compared with the control group (WMD = 0.06, 95% CI: 0.03–0.08, *p* < 0.001) (Figure 2C).

**Figure 3:**
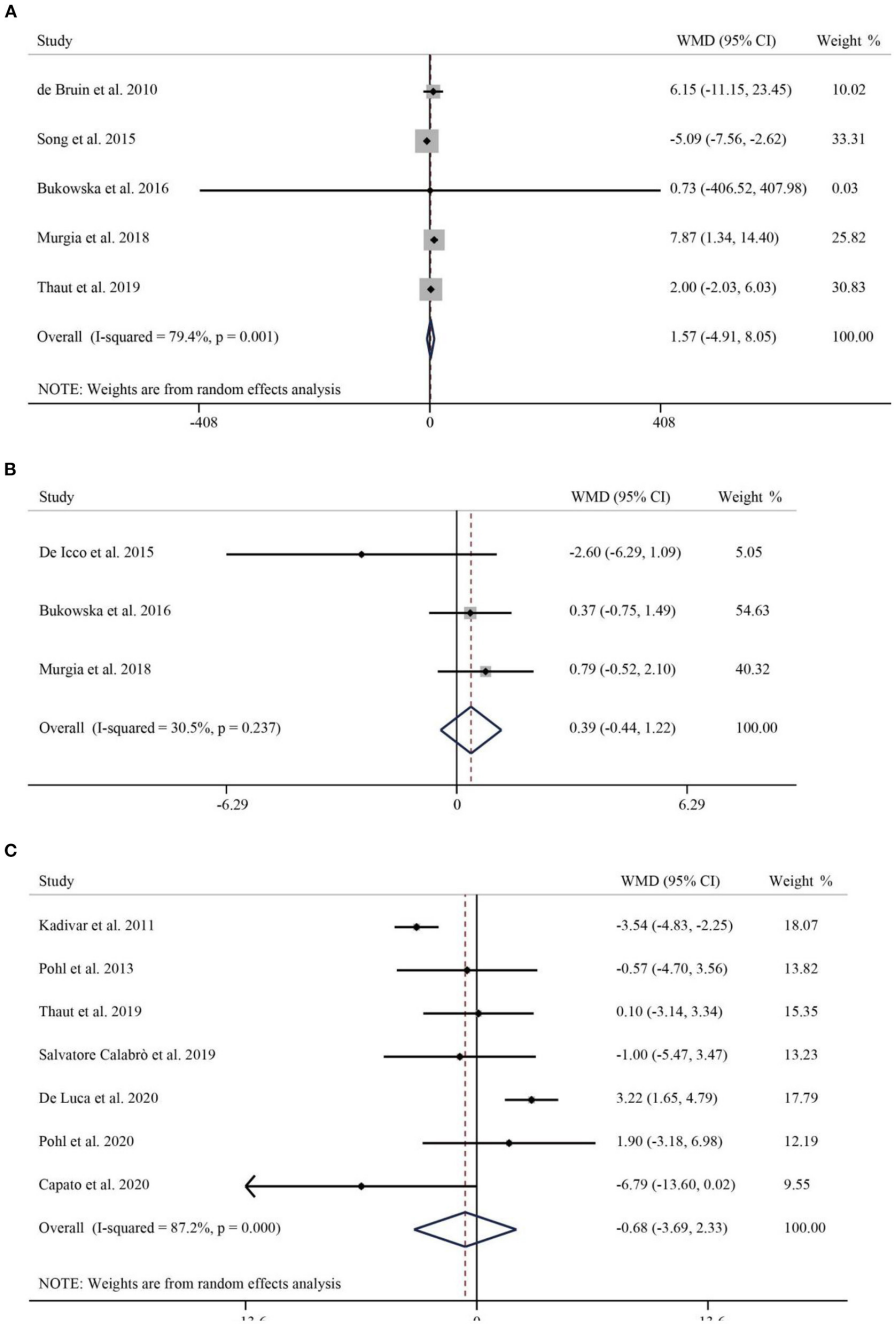
Forest plot of RAS vs. the control group for step frequency **(A)**, swing **(B)**, and TUG **(C)**. TUG, Timed Up-and-Go.

A total of 5 studies with 287 subjects compared patients' step frequency in the intervention and control groups. Due to significant heterogeneity (*I*^2^ = 79.4%, *p* < 0.001), a random-effects model was used to analyze the role of RAS on step frequency. The pooled WMD was 1.57 (95% CI: −4.91–8.05, *P* = 0.635), suggesting that the effect of RAS on step frequency was not significant (Figure 3A). A total of 3 publications reported the percentage of patients swinging. Pooled results showed no statistically significant difference between the rhythmic stimulation and the swing of patients in the control group (WMD = 0.39, 95% CI: −0.44–1.22, *p* = 0.468) (Figure 3B). Seven studies involving 332 Parkinson's patients reported TUG. *I*^2^ = 87.2%, *p* < 0.001, suggesting significant heterogeneity, so a random-effects model was used for the pooled analysis of TUG. There was no significant difference in the effect of RAS in reducing TUG compared to the control group (WMD = −0.68, 95% CI: −3.69–2.33, *P* = 0.658) (Figure 3C).

### Effect of RAS on clinical parameters

**Figure 4:**
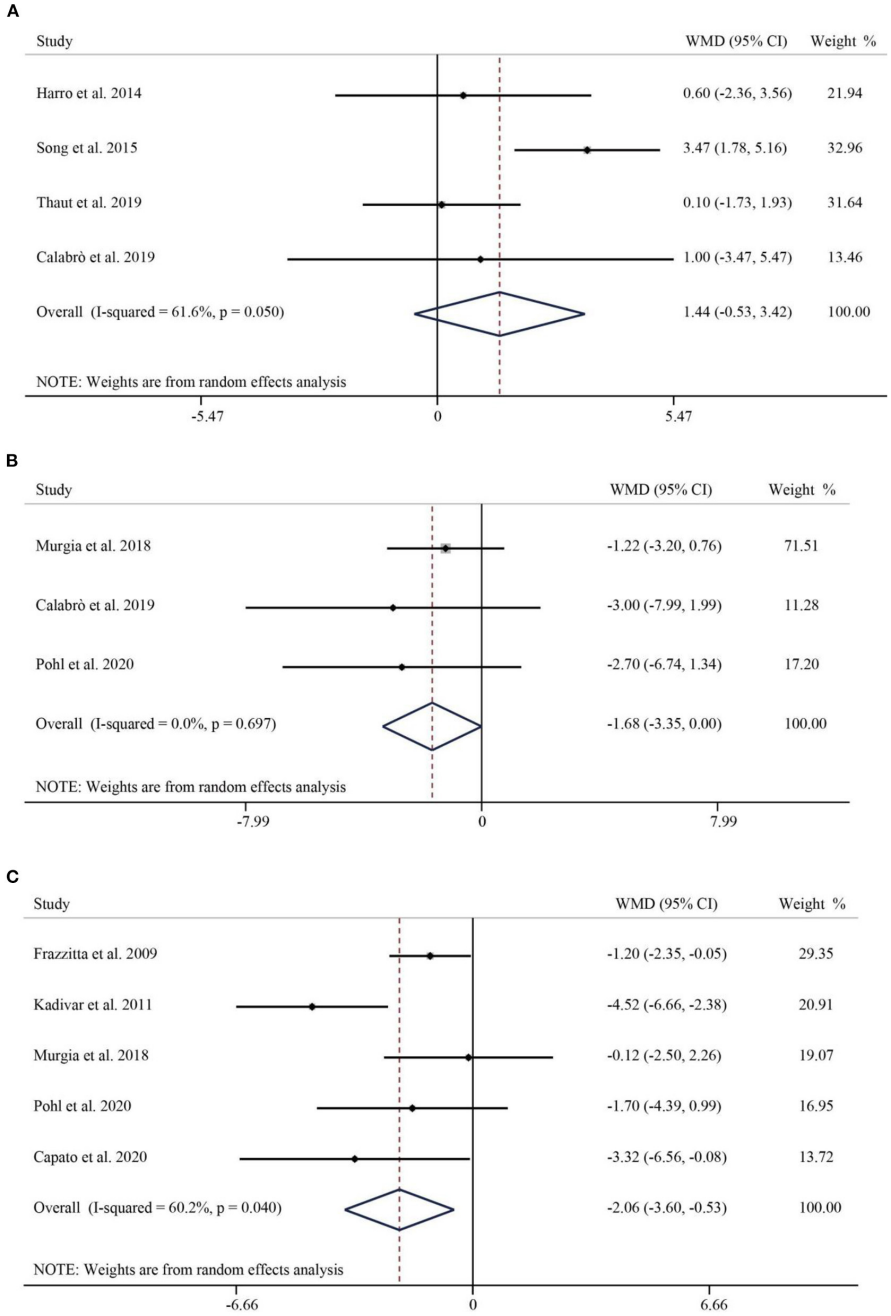
Forest plot of RAS vs. the control group for BBS **(A)**, FES **(B)**, and FOGQ **(C)**. BBS, Berg Balance Scale; FES, Falls Efficacy Scale; FOGQ, Freezing of Gait Questionnaire.

BBS was used to assess balancing capacity of PD patients. A total of 4 studies, including 242 patients, reported the effect of RAS on BBS. The pooled results showed no statistically significant difference between RAS and the control group in improving the balance of patients (WMD = 1.44, 95% CI: −0.53–3.42, *p* = 0.152) (Figure 4A). Next, we used the FES to assess patients' fear of falling. A total of 3 publications reported FES. The pooled WMD was −1.68 (95% CI: −3.35–0.00, *P* = 0.05), suggesting that no significant difference emerged between the control and intervention groups in improving the effect of FES (Figure 4B). Finally, the FOGQ was used to assess patients reported freezing events during walking. Five studies follow the effect of RAS on FOGQ in patients with PD showed that RAS significantly reduced the occurrence of freezing events during walking compared with the control group (WMD = −2.06, 95% CI: −3.60–0.53, *p* = 0.009) (Figure 4C).

**Figure 5:**
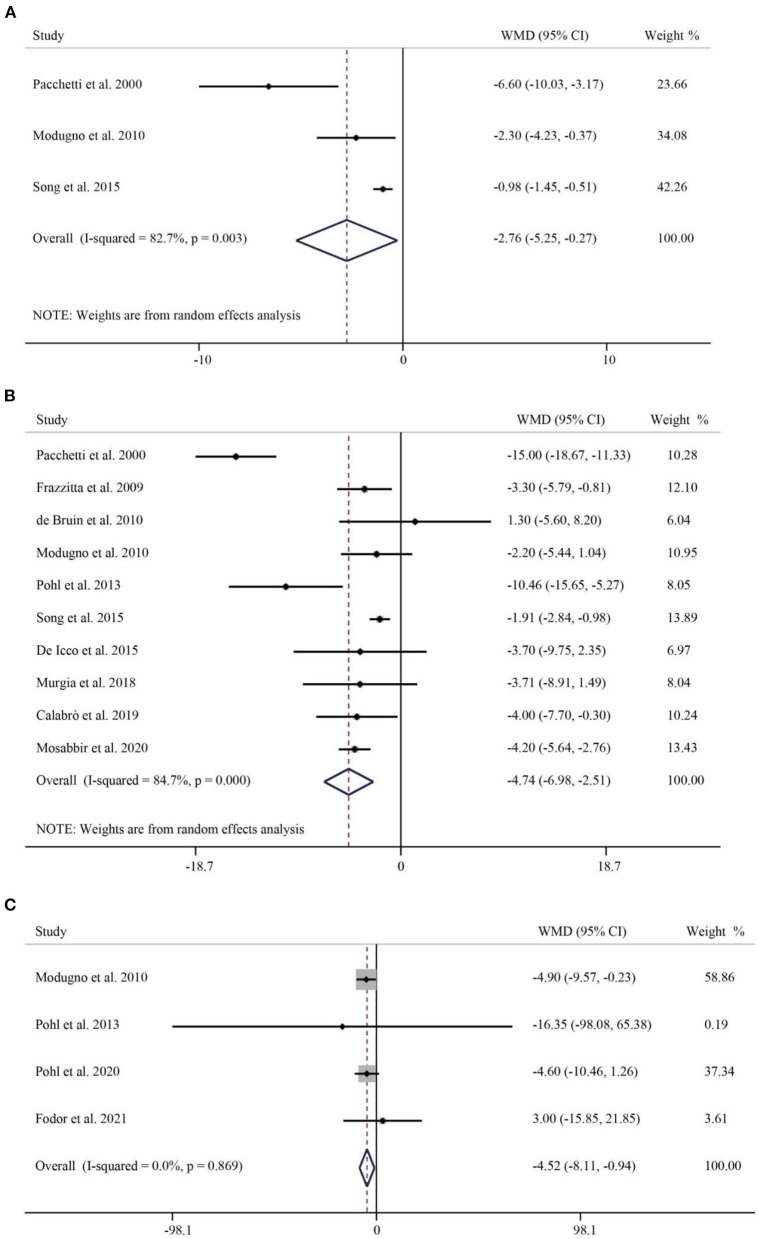
Forest plot of RAS vs. the control group for UPDRS-II **(A)**, UPDRS-III **(B)**, and PDQL **(C)**. UPDRS, Unified Parkinson's Disease Rating Scale; UPDRS-II, UPDRS- Activities of Daily Living; UPDRS-III, UPDRS- Motor Symptoms; PDQL, Parkinson's Disease Quality of Life Questionnaire.

The results obtained in the analysis of the second part of the UPDRS (UPDRS-II) showed that RAS significantly improved impairment in activities of daily living in Parkinson's patients (WMD = −2.76, 95% CI: −5.25 to −0.27, *p* = 0.030) (Figure 5A). The third part of the UPDRS (UPDRS-III) was used to measure motor impairment. A total of 10 studies containing 403 subjects reported the UPDRS-III. The pooled WMD was −4.74 (95% CI: −6.98–2.51, *p* < 0.001), indicating that the RAS significantly reduced the occurrence of dyskinesia with significant heterogeneity (*I*^2^ = 84.7%, *p* < 0.001) (Figure 5B). A total of four papers have examined the effect of RAS on PDQL scores. Compared with the control group, RAS had a positive effect in improving PDQL scores without significant heterogeneity (WMD = −4.52, 95% CI: −8.11– −0.94, *P* = 0.009) (Figure 5C).

### Publication bias

**Figure 6:**
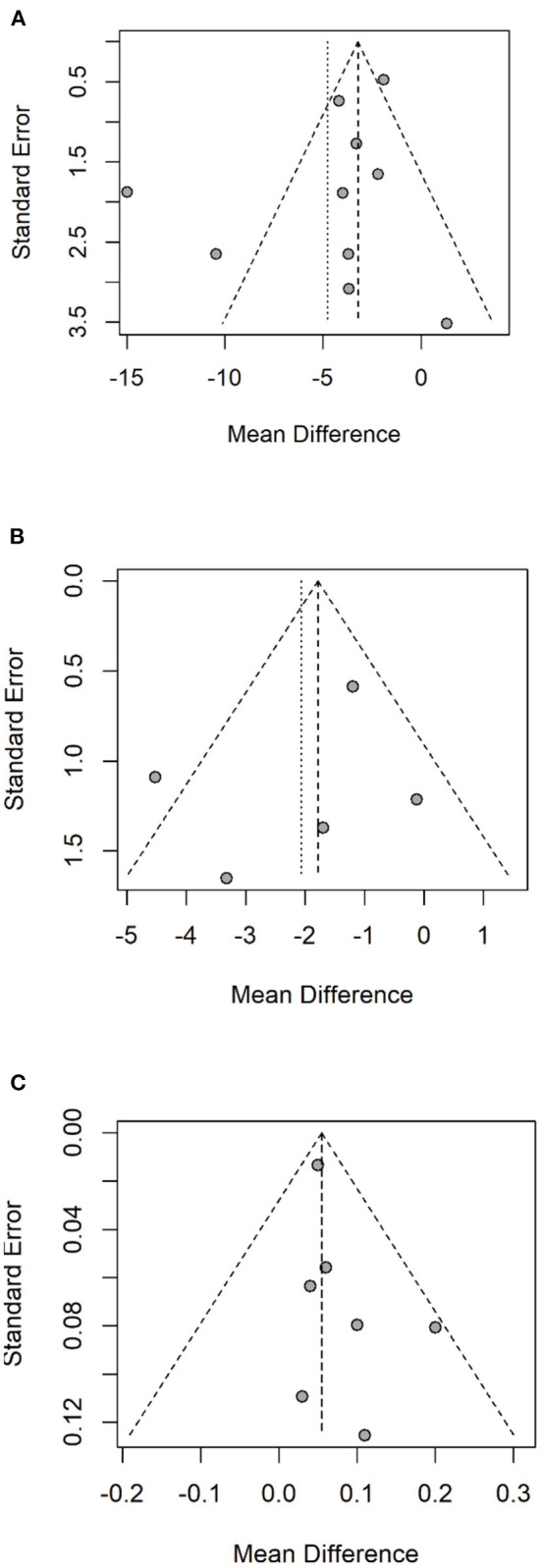
Funnel plot of UPDRS-III **(A)**, FOGQ **(B)**, Speed **(C)**. UPDRS-III, Unified Parkinson's Disease Rating Scale - Motor Symptoms; FOGQ, Freezing of Gait Questionnaire.

The R software was employed to test the publication bias of one primary and two secondary outcomes. These data points represented by individual studies in a funnel plot (Figure 6) were distributed on both sides of the middle solid line, basically in a symmetrical shape. The funnel plots for UPDRS-III, Speed and FOGQ suggest no significant publication bias.

## Discussion

Parkinson's disease is a common age-related neurodegenerative disease and has remained a challenging health problem. Most patients will develop disabling symptoms such as gait freezing, despite optimal medical and surgical therapies. Gait training represent a potentially effective aid for managing PD symptoms not responding to dopaminergic drugs, as cues seem to be able to access rhythmic entrainment mechanisms even in the absence of dopaminergic stimulation (7, 38). This current systematic review and meta-analysis summarizing the effects of the 18 selected studies that met the inclusion criteria, generated the pooled results of RAS exhibiting a significant improvement for gait disturbances, motor activities, and quality of life. In addition, concurrent RAS during physiotherapy significantly increased stride length, accelerated stride speed, reduced the occurrence of walking freezes, promoted mobility, and improved PDQL scores in Parkinson's patients.

Moreover, external stimuli such as acoustic, visual, and somatosensory stimuli can modulate motor patterns in Parkinson's patients, helping them start physical activity and maintain the motivation for motor tasks (19, 38, 46). PD can severely affect patient's gait parameters, such as stride length, stride duration, speed, and gait frequency. The temporal and spatial parameters of gait are associated with unhealthy events in the elderly, including falls, functional decline, and even death (47). Studies have shown that providing RAS alongside gait training significantly improved patients' overall gait quality index, balance, strides length and number, consistent with the results of our meta-analysis (22). The pooled results indicated that RAS had no significant effect on cadence in PD patients. The increase or decrease in step frequency had different effects on patients at different stages of the disease (48). Studies have shown that in order to maintain gait speed, people's gait frequency increases with age. However, the increase in gait frequency can adversely affect the stability of walking (49).

Freezing of gait (FoG), defined as ”a brief, intermittent absence or significant reduction in the forward progress of the foot despite intentional walking," is the most distinctive features of patients with advanced Parkinson's disease (50, 51). FoG can lead to reduced mobility, increased incidence of falls, and a significant negative impact on quality of life (52, 53). Wroblewska et al. found that Nordic walking has a lasting improvement effect on PD patients (54). Studies have shown that receiving auditory and visual cues during treadmill training has a better effect on improving gait freezing than traditional treatments (44). Capato et al. proved that compared with conventional training, RAS can have a significant improvement in the overall well-being of PD during the 6-month follow-up (34).

UPDRS is used to measure the severity of Parkinson's disease. Although UPDRS-II does not directly evaluate the walking and mobility of PD patients, it covers the evaluation of the patient's motor and non-motor symptoms, such as walking, mobility, and other activities of daily living. The results of existing studies and this meta-analysis show that RAS intervention significantly improves UPDRS-II (37, 42, 45). On the other hand, UPDRS-III is used to assess motor status, including tremor, rigidity, bradykinesia, gait, and postural instability. Duncan et al. concluded that compared with the control group, tango can significantly improve the UPDRS-III score of PD patients, and it still has a lasting improvement effect after 12 months of follow-up (55). Our pooled results align with previous studies that RAS significantly reduces the disability scores of UPDRS-III (22, 37, 38).

Levodopa, dopamine agonists, and type B monoamine oxidase inhibitors are traditional medications for Parkinson's disease (9). However, pharmacotherapy can only alleviate symptoms, not the underlying pathology (56). In addition, adverse effects such as loss of potency and toxicity may occur with long-term use of dopaminergic drugs, which may be due to a decrease in the integrity of dopamine transport in the striatal nerve endings of the substantia nigra associated with levodopa. Also, the progression of the disease may reduce the effectiveness of the drug (10, 57). Therefore, RAS has increasingly received attention to enhance gait performance in patients with Parkinson's disease. Rhythmic changes are associated with various neurophysiological changes, such as increased activation of neurons in the frontal-occipital network and increased excitability of spinal motor neurons by the reticulospinal pathway (58).

Acceptance of RAS can facilitate motor activation patterns by increasing frontal-occipital network connectivity and beta frequency oscillations in the cortex (59). Both the basal ganglia and cerebellum influence cortical movement and movement-related areas *via* the thalamus (60, 61). Literatures suggest that the cerebellar-thalamocortical motor network can compensate for the deleterious basal ganglia connection-thalamocortical motor network function associated with internal chronotropic processing (62, 63). Stimulating the cerebellum using oscillating transcranial currents delivered at frequencies similar to intrinsic musical rhythms can largely shape the frontal-parietal connections and the sensorimotor rhythms associated with fine adjustment of gait parameters (22, 64). Thus, the cerebellum may participate in internal timing mechanisms when subjected to external rhythmic auditory stimulation.

The literature included in this meta-analysis was all RCTs, which significantly reduced various potential biases and provided high quality evidence. In addition, various parameters and scales assessing gait, mobility, and quality of life in PD patients were included to evaluate the effectiveness of RAS in improving patients' gait and mobility impairment from multiple aspects.

Limitations of this study should be discussed as they may limit the extrapolation of results. First, the majority of the subjects had mild or moderate disease. This lack of information regarding disease severity and their specific deficits have limited their interpretation of outcomes. Second, the number of studies included in the meta-analysis was limited, and the sample size was small. Only two studies had more than 100 subjects, thirteen studies had <50 subjects, making the generalizability of the study survey difficult. Thirdly, by employing the 18 RCTs we had included in this meta-analysis for the PICOs (Population, Intervention, Control and Outcome evaluation), the Table 2 so constructed has demonstrated for each study the specific intervention method: the interventions given to patients in these 18 individual studies were either RAS, (22, 31, 34–39, 41, 44), Rhythm with Musical melody (23, 30, 31, 33, 40, 42, 43, 45) or Physiotherapy on an Acoustic Vibration Chair (32). For control groups, patients would either receive conventional physical therapy, with or without a structured instruction, or an intervention placebo (the vibration chair without rhythm or melody.) These differences in intervention and control could make the comparison's interpretation difficult. Finally, Language bias has always been possible in meta-analysis. Although all 18 RCTs were published in the English language peer-reviewed journals, the minority (5/18) were from native English-speaking countries. Among all these 18 studies: there were 7 studies from Italy, 1 from Poland, 1 from Romania, 2 from Sweden, 3 from the United States, 2 from Canada, 1 from China and 1 from Brazil. Our Funnel Plots using the UPDRS-III, FoG and Speed as the three major outcomes assessments did not exhibit significant publication biases (Figures 6A–C). However, we are reassured by a recent epidemiology paper by Nussbaumer-Streit 2019 (65) using 59 Cochrane Reviews with or without excluding non-English studies to answer this specific question: excluding non-English publication from evidence syntheses does not change conclusions. In summary, the results of this meta-analysis provide more convincing evidence for the effectiveness of RAS in the rehabilitation of PD patients.

**Table 2:** PICOs (population, intervention, control, outcome and strategy) characteristics of the studies included in the meta-analysis.

**References**	**Population**	**Intervention**	**Comparison**	**Outcome**	**Study**
Pacchetti et al. (45)	Parkinson's disease patients with stable response to levodopa and in Hoehn and Yahr stage 2 or 3	Choral singing, voice exercise, rhythmic and free body movements, and active music involving collective invention	A series of passive stretching exercises, specific motor tasks, and strategies to improve balance and gait	UPDRS-II, UPDRS-III, self-administered HM, and PDQL	RCT
Frazzitta et al. (44)	Patients with a diagnosis of “clinically probable” idiopathic Parkinson's disease	Treadmill training associated with auditory and visual cues	Traditional rehabilitation protocol using only auditory and visual cues	UPDRS III, FOGQ, 6MWT, gait speed, and stride cycle	RCT
de Bruin et al. (43)	Patients with mild to moderate Parkinson's disease	Home training with individual music playlist	Home training with no music	Gait velocity, stride time, stride length, cadence, and UPDRS-III	RCT
Modugno et al. (42)	Patients affected by a moderate form of idiopathic Parkinson's disease	Theater workshop rehabilitation program including vocal music, different emotional moods, performance and physical activities	Physiotherapy Rehabilitation Program	UPDRS, PDQ-39, ESS, SES, and HDRS	RCT
Kadivar et al. (41)	Patients with idiopathic Parkinson's disease	Performed externally paced stepping with rhythmic auditory stimulation	Performed internally paced stepping without rhythmic auditory stimulation	DGI, UPDRS, TUG, and FOGQ	RCT
Pohl et al. (23)	Parkinson's disease patients	Ronnie Gardiner Rhythm and Music Method	Routine drug treatment	UPDRS, SES, PLM, TUG, PDQ-39, CAB, and SDMT	RCT
Harro et al. (40)	Patients with idiopathic Parkinson's disease	Utilized auditory-cued, overground locomotor training on an indoor track while listening to a personalized music playlist set	Utilized moderate intensity treadmill locomotor training with a safety harness	FGS, 6MWT, RST, BBS, LOS, MCT, SOT, fall incidence, ABC-16, and PDQ-39	RCT
Song et al. (37)	Patients with Parkinson's disease	Conventional drug treatment with sound rhythm metronome released as well as the ground fixed ribbon rhythmic visual stimulation walking training	Routine drug treatment with no music	UPDRS-II, UPDRS-III, BBS, and 6MWT	RCT
De Icco et al. (38)	Patients with idiopathic Parkinson's disease	Walking in the presence of rhythmical sounds, or walking on stripes of contrasting color with respect to the floor	Overground training without cues	Gait parameters, gait speed, stride length, UPDRS-III, and FIM	RCT
Bukowska et al. (39)	Patients with idiopathic Parkinson's disease	Daily living, balance, pre-gait and gait training by using sensorimotor NMT techniques (TIMP, PSE, and RAS)	Asked to maintain their daily life activities (changing of position, walking, walking stairs)	Temporal and spatial gait parameters (stance and swing phase, double support, stride time and cadence, step and stride length, velocity and step width)	RCT
Murgia et al. (36)	Patients with Parkinson's disease	Rehabilitation program with ecological RAS 45 min/session, 2/w+3/w home training *5 w; 12 weeks of daily home training	Rehabilitation program with artificial RAS 45 min/session, 2/w+3/w home training *5 w; 12 weeks of daily home training	Spatio-temporal parameters of gait, UPDRS, FIM, SPPB, GDS, PDQ-8, FES, FOGQ, cadence, and gait speed	RCT
Thaut et al. (35)	Patients with idiopathic Parkinson's disease	Completed 24 weeks of RAS training	Discontinued RAS training between weeks 8 and 16	Velocity, stride length, cadence, ankle dorsiflexion, BBS, TUG, FES, and Fall Index	RCT
Calabro et al. (22)	Patients with idiopathic Parkinson's disease	Treadmill training with rhythmic auditory stimulation	Treadmill gait training without rhythmic auditory stimulation	FES, FGA, TUG, UPDRS, gait parameters, and electrophysiological effects	RCT
De Luca et al. (33)	Patients with Parkinson's disease	Treadmill gait training with music therapy	Traditional over ground gait training	PGWBI, Brief- COPE, FIM, TUG, and 10 mWT	RCT
Pohl et al. (31)	Patients with Parkinson's disease	Soft stretching movements, breathing exercises, and exercises typical for the Ronnie Gardiner Method	Usual care without competing activity	TUG, MCAS, SCWT, SDMT, FES, FOGQ, and PDQ-39	RCT
Mosabbir et al. (32)	Patients with Parkinson's disease	40-Hz Physioacoustic Vibrations	Placebo with current levels of physical activity	UPDRS-III, tremor, rigidity, bradykinesia, and posture and gait measures	RCT
Capato et al. (34)	Patients with Parkinson's disease	RAS-supported multimodal balance training	Received no functional balance or gait training	Mini-BESTest, TUG, and NFOG-Q	RCT
Fodor et al. (30)	Patients with idiopathic Parkinson's disease	Multimodal rehabilitation program with music exposure	Same rehabilitation program without music exposure	PDQ-39	RCT

*RAS, rhythmic auditory stimulation; UPDRS, Unified Parkinson's Disease Rating Scale; HM, Happiness Measure; PDQL, Parkinson's Disease Quality of Life Questionnaire; 6MWT, 6-minute walking test; PDQ-39, Parkinson's Disease Quality of Life; ESS, Epworth Sleepiness Scale; SES, The Schwab and England Scale; HDRS, Hamilton Depression Rating Scale; DGI, Dynamic Gait Index; TUG, Timed Up-and-Go; FOGQ, Freezing of Gait Questionnaire; PLM, Posturo-Locomotion-Manual; CAB, Cognitive Assessment Battery; SDMT, the Symbol Digit Modalities Test; BBS, Berg Balance Scale; RST, Rapid Step-Up Test; SOT, NeuroCom Sensory Organization Test; LOS, Limits of Stability; MCT, Motor Control Test; FGS, fast gait speed; ABC-16, Activities-specific Balance Confidence Scale-16; FIM, Functional Independence Measure; SPPB, short physical performance battery; GDS, geriatric depression scale; FES, falls efficacy scale; FGA, Functional Gait Assessment; PGWBI, Psychological General Well-Being Index; Brief- COPE, Brief- Coping Orientation to Problems Experiences; MCAS, Montreal Cognitive Assessment scale; SCWT, Stroop Color-Word Test; SDMT, Symbol Digit Modalities Test; NFOG-Q, New Freezing of Gait Questionnaire; RCT, randomized controlled trial*.

Our study shows the significant efficacy of RAS in improving gait, motor activities and quality of life in Parkinson's patients and suggests its application in clinical practice. However, as the data came from different studies where the sample size, disease severity, stimulation frequency, intervention intensity and functional assessment tools were different. One of the most intriguing examples is the Falls Efficacy Scale (FES). Three out of our 18 studies have independently concluded that rhythmic auditory stimulation can improve gait disturbance (22, 31, 36), however, in combining the original data (a total of 106 patients), the results were of borderline significance (*p* = 0.05). It is desirable to have a multicenter randomized controlled trial that can simultaneously include the key indicators to further determine the RAS efficacy in gait improvement and quality of life in Parkinson's disease.

## Conclusion

In this meta-analysis of 18 randomized controlled trials, we have demonstrated that Rhythmic Auditory Stimulation (RAS) could improve gait, mobility and quality of life in patients with Parkinson's disease. A definitive multi-centre study with a well-defined disease severity, treatment intensity and functional assessment tools should be planned in the future.

## Data availability statement

The original contributions presented in the study are included in the article/supplementary material, further inquiries can be directed to the corresponding author.

## Author contributions

The paper completed at the suggestion and supervised by WP. Two researchers (LL and RH) independently extracted primary data from eligible studies using a standardized form. If the corresponding data could not be extracted directly from the study, it would need to be reanalyzed. Where there were disagreements between above two researchers, a third researcher (XY) was asked to review literatures until a consensus was reached. The data management and statistical analysis were performed by XY and reviewed by statistician YJ from core laboratory. All authors contributed to the article and approved the submitted version.

## Conflict of interest

The authors declare that the research was conducted in the absence of any commercial or financial relationships that could be construed as a potential conflict of interest.

## Publisher's note

All claims expressed in this article are solely those of the authors and do not necessarily represent those of their affiliated organizations, or those of the publisher, the editors and the reviewers. Any product that may be evaluated in this article, or claim that may be made by its manufacturer, is not guaranteed or endorsed by the publisher.

## Abbreviations

RAS: Rhythmic Auditory Stimulation
PD: Parkinson's disease
RCT: Randomized Controlled Trial
PRISMA: Preferred Reporting Items for Systematic Reviews
PEDro: Physiotherapy Evidence Database
TUG: Timed Up-and-Go test
UPDRS: Unified Parkinson's Disease Rating Scale
PDQL: Parkinson's Disease Quality of Life Questionnaire
BBS: Berg Balance Scale
FES: Falls Efficacy Scale
FOGQ: Freezing of Gait Questionnaire
MD: Mean Difference
CI: Confidence Interval
WMD: Weighted Mean Difference
FoG: Freezing of Gait.
